# Crystal structure of 2-oxo-1,2-di­phenyl­ethyl diiso­propyl­carbamate

**DOI:** 10.1107/S2056989021010367

**Published:** 2021-10-13

**Authors:** Viktor Martens, Helmar Görls, Wolfgang Imhof

**Affiliations:** a Institute of Integrated Natural Sciences, University Koblenz - Landau, Universitätsstr. 1, 56070 Koblenz, Germany; b Institute of Inorganic and Analytical Chemistry, Friedrich-Schiller-University Jena, Humboldtstr. 8, 07743 Jena, Germany

**Keywords:** crystal structure, urethanes, carbamates, C—H⋯O hydrogen bonds

## Abstract

The title compound, C_21_H_25_NO_3_, crystallized as a racemic twin in the Sohnke space group *P*2_1_. In the mol­ecular structure of the title compound, both enanti­omers show a highly similar conformation with the urethane function and the benzoyl group showing an almost perpendicular arrangement [the dihedral angle is 72.46 (8)° in the *S*-enanti­omer and 76.21 (8)° in the *R*-enanti­omer]. In the crystal structure, mol­ecules of both enanti­omers show infinite helical arrangements parallel to the *b* axis formed by weak C—H⋯O hydrogen bonds between the phenyl ring of the benzoyl group and the carbamate carbonyl group. In case of the *R*-enanti­omer, this helix is additionally stabilized by a bifurcated hydrogen bond between the carbonyl function of the benzoyl group towards both phenyl groups of the mol­ecule.

## Abstract 2

The title compound, C_21_H_25_NO_3_, crystallizes as a racemic twin in the chiral space group *P*2_1_. Both *R*- and *S*-enanti­omers are connected into infinite helical chains by weak C—H⋯O hydrogen bonds between the phenyl ring of the benzoyl group and the carbamate carbonyl group.

## Chemical context

Phenacyl and desyl compounds may act as photoremovable protecting groups (PPGs) and have been a subject of inter­est for many years (Givens *et al.*, 2012 ▸; Kammari *et al.*, 2007 ▸; Klán *et al.*, 2013 ▸; Sheehan & Umezawa, 1973 ▸). In addition to the protection of carb­oxy­lic acids, they have also been shown to act as suitable groups for the protection and deprotection of amines (Speckmeier *et al.*, 2018 ▸). Besides several carbamate compounds, Lange and co-workers also synthesized the title compound *via* a Cu^I^-catalysed stereospecific coupling reaction using α-stannylated benzyl carbamates (Lange *et al.*, 2008 ▸). We chose a different procedure to synthesize the title compound, according to a synthetic route that has already been reported by Speckmeier *et al.* (2018 ▸). Recently, we reported on the crystal structure of the highly related achiral derivative 2-oxo-2-phenyl­ethyl diiso­propyl­carbamate (Martens *et al.*, 2021 ▸). 
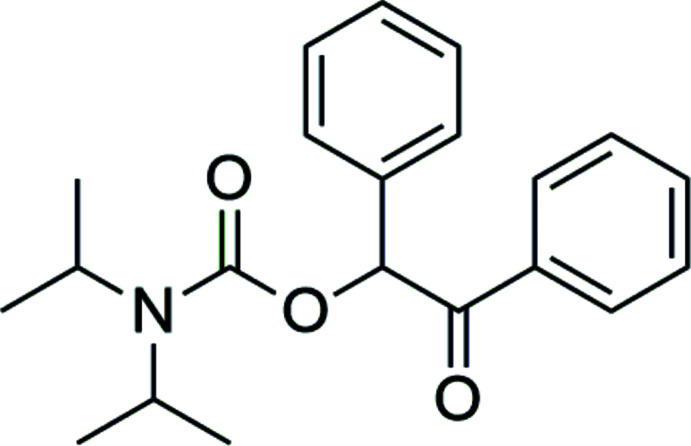


## Structural commentary

**Figure 1:**
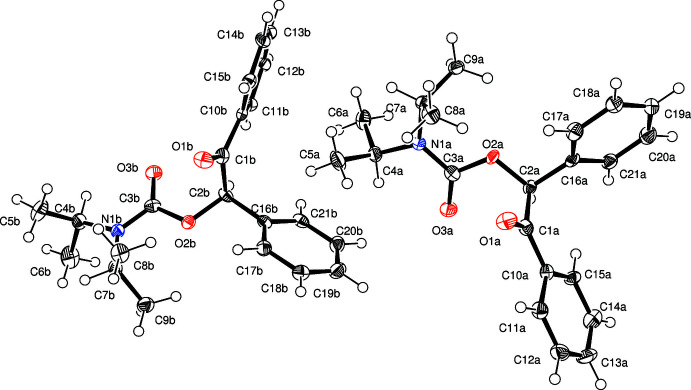
Mol­ecular structures of both enanti­omers of the title compound with displacement ellipsoids drawn at the 50% probability level (*R* left; *S* right).

The carbamate functional moieties (*S*-enanti­omer: N1*A*/C3*A*/O3*A*/O2*A*; *R*-enanti­omer: N1*B*/C3*B*/O3*B*/O2*B*) are essentially planar with the largest deviation for the respective planes being observed for C3*A* and C3*B* (in both cases 0.01 Å). The same is true for the benzoyl groups (*S*-enanti­omer: C1*A*/O1*A*/C10*A*–C15*A*; *R*-enanti­omer: C1*B*/O1*B*/C10*B*–C15*B*). In case of the *S*-enanti­omer, the carbamate and the benzoyl planes subtend a dihedral angle of 77.46 (8)° whereas for the *R*-enanti­omer an angle of 76.21 (8)° is observed (Fig. 1 ▸). These angles show a higher deviation from a perpendicular arrangement than was observed for 2-oxo-2-phenyl­ethyl diiso­propyl­carbamate (Martens *et al.*, 2021 ▸), most probably caused by the enhanced steric requirements of the phenyl substituent at C2*A* or C2*B*, respectively. All other bond lengths and angles are of expected values with C3*A*—N1*A* [1.354 (7) Å], C3*A*—O2*A* [1.360 (7) Å], C3*B*—N1*B* [1.350 (7) Å] and C3*B*—O2*B* [1.363 (6) Å] being slightly shorter than a typical C—O or C—N single bond due to the partial double-bond character of the respective bonds in a carbamate.

## Supra­molecular features

**Figure 2:**
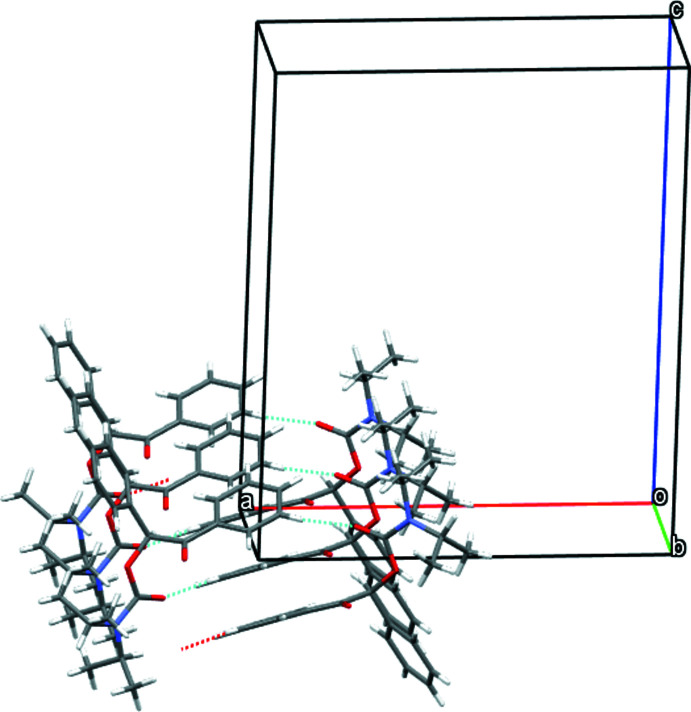
Crystal structure of the *S*-enanti­omer of the title compound showing the helical arrangement of mol­ecules parallel to the *b* axis built up by C—H⋯O hydrogen bonds.

**Figure 3:**
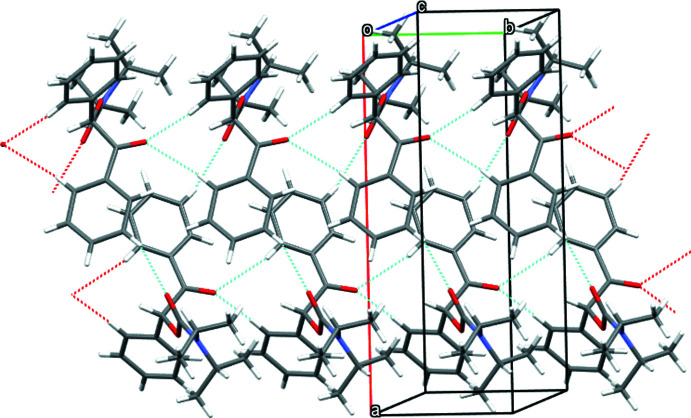
Crystal structure of the *R*-enanti­omer of the title compound showing the helical arrangement of mol­ecules parallel to the *b* axis built up by C—H⋯O hydrogen bonds.

In the crystal structure, mol­ecules of both enanti­omers show infinite helical arrangements parallel to the *b* axis formed by weak C—H⋯O hydrogen bonds (Desiraju & Steiner, 2001 ▸; Figs. 2 ▸ and 3 ▸) between the phenyl ring of the benzoyl group and the carbamate carbonyl group (*S*-enanti­omer: C12*A*—H12*A*⋯O3*A*, *R*-enanti­omer: C14*B*—H14*B*⋯O3*B*; Table 1 ▸). In each of the helices, only one enanti­omer is present. Nevertheless, the helices do not act as mirror images because the arrangement of the mol­ecules relative to each other is different. In the case of the *R*-enanti­omer (Fig. 3 ▸), the supra­molecular helix is additionally stabilized by a bifurcated hydrogen bond between the carbonyl function of the benzoyl group towards both phenyl groups of the mol­ecule (C11*B*—H12*B*⋯O1*B* and C12*B*—H12*B*⋯O1*B*; Table 1 ▸).

## Database survey

In the Cambridge Structural Database (CSD; ConQuest Version 2020.3.0; Groom *et al.*, 2016 ▸) there is only one carbamate reported with a CH_2_—C(O)—Ph group attached to the carbamate oxygen atom (NIWQUI; Jiang *et al.*, 2019 ▸). This compound shows a di­ethyl­amino group and a *p*-chloro­phenyl substituent instead of the diiso­propyl­amino group and the non-substituted phenyl group as in the title compound. Contrary to the title compound, the carbamate plane and the benzoyl plane are almost coplanar. The carbonyl oxygen atoms show numerous short contacts towards different C—H groups of neighbouring mol­ecules, leading to a dense three-dimensional network. In addition, we recently reported a structure, in which there also is a CH_2_—C(O)—Ph group instead of the CH(Ph)—C(O)—Ph unit in the title compound (Martens *et al.*, 2021 ▸). In this structure, a layered arrangement is realized by all three oxygen atoms acting as hydrogen-bond acceptor sites. Moreover, there is one structure reported in the literature that is identical to the title compound with the exception of one bromine substituent at the 4-position of the phenyl ring attached to the C1=O1 carbonyl group (DOKMAS; Lange *et al.*, 2008 ▸). In the latter case, the enanti­opure *S*-enanti­omer was crystallized. The supra­molecular structure of this compound shows the same bifurcated hydrogen bond as is observed for the *R*-enanti­omer of the title compound. On the other hand, the analogue of O3 is not engaged in a C—H⋯O inter­action but shows a short oxygen–bromine contact (3.139 Å). These two inter­actions lead to a double-strand arrangement of mol­ecules parallel to the *a* axis.

## Synthesis and crystallization

Diiso­propyl­amine (0.05 mol, 5.05 g) and one equivalent of caesium carbonate (0.05 mol, 16.55 g) were placed in a Schlenk tube and dissolved in anhydrous DMSO (150 ml). The tube was sealed with a septum, and two balloons filled with CO_2_ were bubbled through the reaction mixture within one h while stirring. After the addition of CO_2_, 1.1 equivalents of 2-bromo-1,2-di­phenyl­ethan-1-one (0.055 mol, 15.13 g) dissolved in a small amount of DMSO were added in one portion. The consumption of the 2-bromo-1,2-di­phenyl­ethan-1-one was monitored by TLC, and after 30 min the reaction mixture was poured onto ice to quench the reaction. After extraction with di­chloro­methane (3 × 40 ml), the combined organic phases were washed with brine, separated and dried over Na_2_SO_4_. The solvent was removed *in vacuo* and the crude product was recrystallized from *n*-hexa­ne/ethyl­acetate (4:1, *v*/*v*) to afford the title compound (16.12 g; 95%) as a colourless crystalline solid. M.p. 485 K; ^1^H NMR (500 MHz, CDCl_3_) [ppm]: δ = 7.96 (*dd*, 2H), 7.50–7.47 (*m*, 3H), 7.39–7.32 (*m*, 5H), 6.88 (*s*, 1H), 4.05 (*s*, 1H), 3.86 (*s*, 1H), 1.28 (*d*, 12H); ^13^C NMR (126 MHz, CDCl_3_) [ppm]: δ = 195.4 (C=O), 154.8 (NC=O), 135.2, 134.5, 133.3, 129.0, 129.0, 128.9, 128.7, 128.7 (C_Ph_), 77.7 (C benzylic), 46.8, 45.9 [(H_3_C)_2_CH–], 21.6, 21.4 [(H_3_C)_2_CH–].

## Refinement

Crystal data, data collection and structure refinement details are summarized in Table 2 ▸. All hydrogen atoms were placed in idealized positions (C—H = 0.95–0.98 Å) and refined using a riding model with isotropic displacement parameters calculated as *U*_iso_(H) = 1.2(C) for methine and hydrogen atoms of the phenyl group or 1.5×*U*_eq_(C) for methyl groups. The crystal studied was refined as a two-component twin with fractions of 29% *vs* 71%.

## Supplementary Material

Crystal structure: contains datablock(s) I. DOI: 10.1107/S2056989021010367/wm5618sup1.cif

Structure factors: contains datablock(s) I. DOI: 10.1107/S2056989021010367/wm5618Isup2.hkl

Supporting information file. DOI: 10.1107/S2056989021010367/wm5618Isup3.cml

CCDC reference: 2114278

Additional supporting information:  crystallographic information; 3D view; checkCIF report

## Figures and Tables

**Table 1:** Hydrogen-bond geometry (Å, °)

*D*—H⋯*A*	*D*—H	H⋯*A*	*D*⋯*A*	*D*—H⋯*A*
C12*A*—H12*A*⋯O3*A* ^i^	0.95	2.36	3.309 (2)	174
C14*B*—H14*B*⋯O3*B* ^ii^	0.95	2.58	3.288 (2)	132
C11*B*—H11*B*⋯O1*B* ^iii^	0.95	2.69	3.553 (2)	152
C21*B*—H21*B*⋯O1*B* ^iii^	0.95	2.62	3.522 (2)	158

Symmetry codes: (i) -x+2, y+{\script{1\over 2}}, -z; (ii) -x+1, y+{\script{1\over 2}}, -z+1; (iii) x, y-1, z.

**Table 2:** Experimental details

Crystal data	
Chemical formula	C_21_H_25_NO_3_
*M*_r_	339.42
Crystal system, space group	Monoclinic, *P*2_1_
Temperature (K)	133
*a*, *b*, *c* (Å)	15.7976 (5), 5.9184 (3), 19.5340 (8)
β (°)	90.310 (2)
*V* (Å^3^)	1826.33 (13)
*Z*	4
Radiation type	Mo *K*α
μ (mm^−1^)	0.08
Crystal size (mm)	0.11 × 0.10 × 0.09

Data collection	
Diffractometer	Nonius KappaCCD
Absorption correction	Multi-scan (*SADABS*; Krause *et al.*, 2015 ▸)
*T*_min_, *T*_max_	0.659, 0.746
No. of measured, independent and observed [*I* > 2σ(*I*)] reflections	18477, 8221, 7092
*R*_int_	0.051
(sin θ/λ)_max_ (Å^−1^)	0.649

Refinement	
*R*[*F* ^2^ > 2σ(*F* ^2^)], *wR*(*F* ^2^), *S*	0.069, 0.140, 1.09
No. of reflections	8221
No. of parameters	460
No. of restraints	1
H-atom treatment	H-atom parameters constrained
Δρ_max_, Δρ_min_ (e Å^−3^)	0.27, −0.25
Absolute structure	Twinning involves inversion, so Flack parameter cannot be determined

Computer programs: *COLLECT* (Nonius 1998 ▸), *DENZO* (Otwinowski & Minor, 1997 ▸), *SHELXT* (Sheldrick, 2015*a* ▸), *SHELXL* (Sheldrick, 2015*b* ▸), *ORTEP-3 for Windows* (Farrugia, 2012 ▸) and *Mercury* (Macrae *et al.*, 2020 ▸).

## supplementary crystallographic information

### Crystal data

**Table:**

C_21_H_25_NO_3_	*F*(000) = 728
*M**_r_* = 339.42	*D*_x_ = 1.234 Mg m^−^^3^
Monoclinic, *P*2_1_	Mo *K*α radiation, λ = 0.71073 Å
*a* = 15.7976 (5) Å	Cell parameters from 18477 reflections
*b* = 5.9184 (3) Å	θ = 1.7–27.5°
*c* = 19.5340 (8) Å	µ = 0.08 mm^−^^1^
β = 90.310 (2)°	*T* = 133 K
*V* = 1826.33 (13) Å^3^	Prism, colourless
*Z* = 4	0.11 × 0.10 × 0.09 mm

### Data collection

**Table:**

Nonius KappaCCD diffractometer	7092 reflections with *I* > 2σ(*I*)
phi + ω – scans	*R*_int_ = 0.051
Absorption correction: multi-scan (*SADABS*; Krause *et al.*, 2015)	θ_max_ = 27.5°, θ_min_ = 1.7°
*T*_min_ = 0.659, *T*_max_ = 0.746	*h* = −20→20
18477 measured reflections	*k* = −7→7
8221 independent reflections	*l* = −25→25

### Refinement

**Table:**

Refinement on *F*^2^	Secondary atom site location: difference Fourier map
Least-squares matrix: full	Hydrogen site location: inferred from neighbouring sites
*R*[*F*^2^ > 2σ(*F*^2^)] = 0.069	H-atom parameters constrained
*wR*(*F*^2^) = 0.140	*w* = 1/[σ^2^(*F*_o_^2^) + 2.2197*P*] where *P* = (*F*_o_^2^ + 2*F*_c_^2^)/3
*S* = 1.09	(Δ/σ)_max_ < 0.001
8221 reflections	Δρ_max_ = 0.27 e Å^−^^3^
460 parameters	Δρ_min_ = −0.25 e Å^−^^3^
1 restraint	Absolute structure: Twinning involves inversion, so Flack parameter cannot be determined
Primary atom site location: structure-invariant direct methods	

### Special details

**Table:**

Geometry. All esds (except the esd in the dihedral angle between two l.s. planes) are estimated using the full covariance matrix. The cell esds are taken into account individually in the estimation of esds in distances, angles and torsion angles; correlations between esds in cell parameters are only used when they are defined by crystal symmetry. An approximate (isotropic) treatment of cell esds is used for estimating esds involving l.s. planes.
Refinement. Refined as a two-component inversion twin.

### Fractional atomic coordinates and isotropic or equivalent isotropic displacement parameters (Å^2^)

**Table:**

	*x*	*y*	*z*	*U*_iso_*/*U*_eq_	
O1A	0.8121 (3)	0.6853 (7)	−0.0240 (2)	0.0288 (10)	
O2A	0.6870 (3)	0.4176 (7)	0.01863 (19)	0.0190 (9)	
O3A	0.7822 (3)	0.2958 (8)	0.0975 (2)	0.0261 (10)	
N1A	0.6619 (3)	0.4852 (9)	0.1293 (2)	0.0199 (11)	
C1A	0.8205 (4)	0.4889 (10)	−0.0388 (3)	0.0194 (12)	
C2A	0.7421 (3)	0.3312 (10)	−0.0333 (3)	0.0170 (11)	
H2A	0.759703	0.172549	−0.022792	0.020*	
C3A	0.7164 (4)	0.3938 (10)	0.0838 (3)	0.0201 (12)	
C4A	0.6799 (4)	0.4475 (11)	0.2031 (3)	0.0224 (13)	
H4A	0.731816	0.351359	0.206096	0.027*	
C5A	0.6988 (4)	0.6660 (12)	0.2408 (3)	0.0337 (16)	
H5AA	0.717040	0.631644	0.287699	0.051*	
H5AB	0.647681	0.759697	0.242005	0.051*	
H5AC	0.743922	0.747829	0.217106	0.051*	
C6A	0.6084 (4)	0.3161 (12)	0.2359 (3)	0.0286 (14)	
H6AA	0.625444	0.268598	0.281976	0.043*	
H6AB	0.595400	0.182491	0.208083	0.043*	
H6AC	0.557999	0.412251	0.238758	0.043*	
C7A	0.5909 (3)	0.6403 (10)	0.1106 (3)	0.0186 (12)	
H7A	0.563719	0.685615	0.154630	0.022*	
C8A	0.6230 (4)	0.8573 (11)	0.0779 (3)	0.0267 (13)	
H8AA	0.666444	0.925211	0.107349	0.040*	
H8AB	0.575809	0.963387	0.072233	0.040*	
H8AC	0.647270	0.822968	0.033024	0.040*	
C9A	0.5215 (4)	0.5267 (11)	0.0676 (3)	0.0218 (12)	
H9AA	0.471118	0.623236	0.066660	0.033*	
H9AB	0.507155	0.380250	0.087855	0.033*	
H9AC	0.541918	0.504067	0.020820	0.033*	
C10A	0.9019 (4)	0.3949 (9)	−0.0646 (3)	0.0172 (11)	
C11A	0.9726 (4)	0.5377 (11)	−0.0640 (3)	0.0238 (13)	
H11A	0.967778	0.685807	−0.045748	0.029*	
C12A	1.0490 (4)	0.4657 (12)	−0.0897 (3)	0.0303 (15)	
H12A	1.096641	0.563704	−0.088757	0.036*	
C13A	1.0564 (4)	0.2506 (13)	−0.1167 (3)	0.0327 (16)	
H13A	1.108798	0.202773	−0.135411	0.039*	
C14A	0.9876 (4)	0.1038 (11)	−0.1168 (3)	0.0297 (15)	
H14A	0.993042	−0.044172	−0.135173	0.036*	
C15A	0.9104 (3)	0.1759 (10)	−0.0897 (3)	0.0213 (12)	
H15A	0.863640	0.075140	−0.088342	0.026*	
C16A	0.6918 (3)	0.3416 (10)	−0.0996 (3)	0.0184 (12)	
C17A	0.6459 (4)	0.5366 (11)	−0.1152 (3)	0.0253 (13)	
H17A	0.647648	0.661921	−0.084779	0.030*	
C18A	0.5975 (4)	0.5490 (12)	−0.1749 (3)	0.0276 (14)	
H18A	0.565708	0.681533	−0.184613	0.033*	
C19A	0.5955 (4)	0.3686 (12)	−0.2201 (3)	0.0294 (15)	
H19A	0.562904	0.377176	−0.261009	0.035*	
C20A	0.6413 (4)	0.1766 (12)	−0.2050 (3)	0.0302 (15)	
H20A	0.639871	0.052366	−0.235793	0.036*	
C21A	0.6895 (4)	0.1619 (11)	−0.1456 (3)	0.0230 (13)	
H21A	0.721040	0.028715	−0.136229	0.028*	
O1B	0.6967 (3)	0.7307 (7)	0.4533 (2)	0.0271 (10)	
O2B	0.8103 (3)	0.4526 (7)	0.51374 (18)	0.0194 (9)	
O3B	0.7098 (3)	0.3500 (8)	0.5898 (2)	0.0264 (10)	
N1B	0.8295 (3)	0.5399 (9)	0.6247 (2)	0.0196 (10)	
C1B	0.6829 (4)	0.5301 (10)	0.4479 (3)	0.0200 (12)	
C2B	0.7551 (3)	0.3627 (10)	0.4613 (3)	0.0183 (12)	
H2B	0.732304	0.212046	0.475242	0.022*	
C3B	0.7777 (4)	0.4423 (10)	0.5781 (3)	0.0189 (12)	
C4B	0.8101 (4)	0.5094 (11)	0.6976 (3)	0.0258 (14)	
H4B	0.756939	0.418041	0.700236	0.031*	
C5B	0.7933 (5)	0.7329 (14)	0.7337 (3)	0.0413 (19)	
H5BA	0.846412	0.817367	0.738191	0.062*	
H5BB	0.770122	0.703504	0.779273	0.062*	
H5BC	0.752597	0.821747	0.706896	0.062*	
C6B	0.8799 (5)	0.3745 (13)	0.7337 (3)	0.0386 (18)	
H6BA	0.863578	0.346188	0.781268	0.058*	
H6BB	0.932837	0.460894	0.732922	0.058*	
H6BC	0.888072	0.230129	0.710081	0.058*	
C7B	0.9040 (4)	0.6810 (10)	0.6067 (3)	0.0216 (12)	
H7B	0.928159	0.734045	0.651270	0.026*	
C8B	0.8777 (4)	0.8945 (11)	0.5683 (3)	0.0278 (14)	
H8BA	0.833198	0.972502	0.593895	0.042*	
H8BB	0.856216	0.853703	0.522793	0.042*	
H8BC	0.926743	0.994531	0.563576	0.042*	
C9B	0.9745 (4)	0.5502 (11)	0.5720 (3)	0.0272 (14)	
H9BA	0.986965	0.413125	0.598401	0.041*	
H9BB	1.025404	0.644571	0.569792	0.041*	
H9BC	0.956746	0.508404	0.525597	0.041*	
C10B	0.5976 (3)	0.4428 (10)	0.4260 (3)	0.0165 (11)	
C11B	0.5695 (4)	0.2237 (10)	0.4395 (3)	0.0206 (12)	
H11B	0.606805	0.115534	0.459205	0.025*	
C12B	0.4860 (3)	0.1654 (11)	0.4238 (3)	0.0223 (12)	
H12B	0.466538	0.016660	0.433074	0.027*	
C13B	0.4313 (4)	0.3211 (12)	0.3951 (3)	0.0260 (14)	
H13B	0.374611	0.278911	0.384894	0.031*	
C14B	0.4588 (4)	0.5393 (11)	0.3809 (3)	0.0232 (13)	
H14B	0.421022	0.646333	0.361192	0.028*	
C15B	0.5419 (4)	0.5997 (10)	0.3959 (3)	0.0221 (13)	
H15B	0.561242	0.747893	0.385732	0.027*	
C16B	0.8076 (3)	0.3419 (10)	0.3967 (3)	0.0185 (11)	
C17B	0.8615 (4)	0.5147 (11)	0.3770 (3)	0.0220 (12)	
H17B	0.866476	0.646580	0.404392	0.026*	
C18B	0.9080 (4)	0.4954 (11)	0.3172 (3)	0.0235 (13)	
H18B	0.944589	0.614749	0.303810	0.028*	
C19B	0.9015 (4)	0.3042 (12)	0.2771 (3)	0.0286 (15)	
H19B	0.933510	0.292162	0.236173	0.034*	
C20B	0.8484 (4)	0.1302 (11)	0.2965 (3)	0.0259 (14)	
H20B	0.843657	−0.001200	0.268859	0.031*	
C21B	0.8016 (4)	0.1475 (10)	0.3570 (3)	0.0217 (12)	
H21B	0.765911	0.026904	0.370783	0.026*	

### Atomic displacement parameters (Å^2^)

**Table:**

	*U*^11^	*U*^22^	*U*^33^	*U*^12^	*U*^13^	*U*^23^
O1A	0.026 (2)	0.018 (2)	0.042 (3)	−0.0023 (19)	0.005 (2)	−0.007 (2)
O2A	0.019 (2)	0.022 (2)	0.0166 (18)	0.0056 (17)	0.0047 (16)	−0.0037 (16)
O3A	0.023 (2)	0.029 (3)	0.027 (2)	0.0088 (19)	−0.0014 (18)	0.0011 (18)
N1A	0.017 (2)	0.026 (3)	0.017 (2)	0.004 (2)	−0.0009 (18)	−0.001 (2)
C1A	0.023 (3)	0.015 (3)	0.020 (3)	−0.001 (2)	−0.002 (2)	−0.002 (2)
C2A	0.020 (3)	0.015 (3)	0.016 (2)	0.005 (2)	0.002 (2)	0.000 (2)
C3A	0.022 (3)	0.015 (3)	0.023 (3)	−0.003 (2)	−0.002 (2)	0.001 (2)
C4A	0.024 (3)	0.024 (3)	0.019 (3)	0.006 (3)	−0.003 (2)	−0.003 (2)
C5A	0.037 (4)	0.040 (4)	0.025 (3)	−0.008 (3)	−0.009 (3)	−0.007 (3)
C6A	0.039 (4)	0.026 (3)	0.021 (3)	−0.005 (3)	0.003 (3)	0.000 (3)
C7A	0.017 (3)	0.023 (3)	0.015 (3)	0.006 (2)	0.000 (2)	0.001 (2)
C8A	0.032 (3)	0.018 (3)	0.029 (3)	0.002 (3)	−0.003 (3)	−0.002 (3)
C9A	0.018 (3)	0.025 (3)	0.022 (3)	0.003 (3)	0.002 (2)	0.001 (3)
C10A	0.023 (3)	0.013 (3)	0.015 (3)	−0.001 (2)	0.000 (2)	0.003 (2)
C11A	0.026 (3)	0.021 (3)	0.024 (3)	−0.004 (3)	0.000 (2)	−0.001 (3)
C12A	0.024 (3)	0.037 (4)	0.030 (3)	−0.009 (3)	0.000 (3)	0.002 (3)
C13A	0.019 (3)	0.045 (4)	0.034 (4)	0.008 (3)	0.008 (3)	0.010 (3)
C14A	0.035 (3)	0.026 (4)	0.029 (3)	0.010 (3)	0.006 (3)	0.008 (3)
C15A	0.017 (3)	0.024 (3)	0.023 (3)	−0.002 (2)	0.005 (2)	0.006 (2)
C16A	0.016 (3)	0.020 (3)	0.019 (3)	−0.007 (2)	0.008 (2)	−0.001 (2)
C17A	0.029 (3)	0.021 (3)	0.026 (3)	−0.001 (3)	0.003 (3)	−0.002 (3)
C18A	0.026 (3)	0.030 (4)	0.026 (3)	0.005 (3)	0.000 (3)	−0.003 (3)
C19A	0.023 (3)	0.044 (4)	0.020 (3)	0.000 (3)	0.000 (2)	−0.004 (3)
C20A	0.029 (3)	0.032 (4)	0.030 (3)	−0.006 (3)	0.004 (3)	−0.012 (3)
C21A	0.022 (3)	0.023 (3)	0.024 (3)	0.004 (3)	0.003 (2)	−0.002 (2)
O1B	0.026 (2)	0.016 (2)	0.039 (3)	−0.0019 (18)	−0.003 (2)	0.0013 (18)
O2B	0.019 (2)	0.022 (2)	0.0173 (18)	−0.0015 (17)	0.0029 (16)	0.0019 (16)
O3B	0.023 (2)	0.029 (2)	0.027 (2)	−0.0082 (19)	0.0045 (17)	0.0007 (19)
N1B	0.020 (2)	0.022 (3)	0.017 (2)	−0.002 (2)	0.0021 (19)	0.000 (2)
C1B	0.024 (3)	0.018 (3)	0.018 (3)	−0.003 (3)	0.003 (2)	0.002 (2)
C2B	0.019 (3)	0.014 (3)	0.022 (3)	−0.002 (2)	−0.007 (2)	0.001 (2)
C3B	0.018 (3)	0.015 (3)	0.024 (3)	0.004 (2)	0.000 (2)	0.006 (2)
C4B	0.025 (3)	0.035 (4)	0.017 (3)	−0.009 (3)	0.004 (2)	0.001 (3)
C5B	0.047 (4)	0.047 (5)	0.030 (4)	0.004 (4)	0.015 (3)	−0.007 (3)
C6B	0.054 (5)	0.039 (5)	0.023 (3)	−0.004 (4)	0.000 (3)	0.011 (3)
C7B	0.025 (3)	0.021 (3)	0.020 (3)	−0.005 (2)	0.001 (2)	−0.003 (2)
C8B	0.034 (3)	0.021 (3)	0.028 (3)	−0.004 (3)	0.000 (3)	0.004 (3)
C9B	0.024 (3)	0.028 (4)	0.030 (3)	−0.002 (3)	0.003 (3)	−0.002 (3)
C10B	0.017 (3)	0.019 (3)	0.013 (2)	0.001 (2)	0.002 (2)	−0.003 (2)
C11B	0.019 (3)	0.021 (3)	0.022 (3)	−0.001 (2)	0.001 (2)	0.004 (2)
C12B	0.020 (3)	0.021 (3)	0.026 (3)	−0.006 (2)	0.002 (2)	−0.003 (3)
C13B	0.019 (3)	0.038 (4)	0.021 (3)	−0.004 (3)	0.002 (2)	−0.006 (3)
C14B	0.023 (3)	0.027 (3)	0.020 (3)	0.006 (3)	−0.006 (2)	0.000 (3)
C15B	0.024 (3)	0.020 (3)	0.022 (3)	0.004 (2)	0.003 (2)	−0.003 (2)
C16B	0.018 (3)	0.018 (3)	0.019 (3)	0.000 (2)	−0.004 (2)	0.003 (2)
C17B	0.023 (3)	0.021 (3)	0.022 (3)	0.003 (3)	−0.001 (2)	0.000 (2)
C18B	0.022 (3)	0.024 (3)	0.024 (3)	−0.001 (3)	0.001 (2)	0.007 (3)
C19B	0.025 (3)	0.037 (4)	0.024 (3)	0.004 (3)	0.005 (3)	0.003 (3)
C20B	0.029 (3)	0.025 (3)	0.024 (3)	0.007 (3)	−0.001 (3)	−0.004 (3)
C21B	0.020 (3)	0.019 (3)	0.026 (3)	0.000 (2)	−0.007 (2)	0.002 (2)

### Geometric parameters (Å, º)

**Table:**

O1A—C1A	1.205 (7)	O1B—C1B	1.212 (7)
O2A—C3A	1.360 (7)	O2B—C3B	1.363 (6)
O2A—C2A	1.435 (6)	O2B—C2B	1.443 (6)
O3A—C3A	1.219 (7)	O3B—C3B	1.227 (7)
N1A—C3A	1.354 (7)	N1B—C3B	1.350 (7)
N1A—C4A	1.484 (7)	N1B—C4B	1.469 (7)
N1A—C7A	1.493 (7)	N1B—C7B	1.486 (7)
C1A—C10A	1.491 (8)	C1B—C10B	1.503 (8)
C1A—C2A	1.555 (8)	C1B—C2B	1.533 (8)
C2A—C16A	1.516 (8)	C2B—C16B	1.519 (8)
C2A—H2A	1.0000	C2B—H2B	1.0000
C4A—C6A	1.516 (8)	C4B—C5B	1.524 (10)
C4A—C5A	1.518 (9)	C4B—C6B	1.531 (9)
C4A—H4A	1.0000	C4B—H4B	1.0000
C5A—H5AA	0.9800	C5B—H5BA	0.9800
C5A—H5AB	0.9800	C5B—H5BB	0.9800
C5A—H5AC	0.9800	C5B—H5BC	0.9800
C6A—H6AA	0.9800	C6B—H6BA	0.9800
C6A—H6AB	0.9800	C6B—H6BB	0.9800
C6A—H6AC	0.9800	C6B—H6BC	0.9800
C7A—C8A	1.523 (8)	C7B—C9B	1.519 (8)
C7A—C9A	1.532 (8)	C7B—C8B	1.526 (8)
C7A—H7A	1.0000	C7B—H7B	1.0000
C8A—H8AA	0.9800	C8B—H8BA	0.9800
C8A—H8AB	0.9800	C8B—H8BB	0.9800
C8A—H8AC	0.9800	C8B—H8BC	0.9800
C9A—H9AA	0.9800	C9B—H9BA	0.9800
C9A—H9AB	0.9800	C9B—H9BB	0.9800
C9A—H9AC	0.9800	C9B—H9BC	0.9800
C10A—C15A	1.392 (8)	C10B—C11B	1.396 (8)
C10A—C11A	1.400 (8)	C10B—C15B	1.405 (8)
C11A—C12A	1.377 (8)	C11B—C12B	1.396 (8)
C11A—H11A	0.9500	C11B—H11B	0.9500
C12A—C13A	1.384 (10)	C12B—C13B	1.380 (9)
C12A—H12A	0.9500	C12B—H12B	0.9500
C13A—C14A	1.391 (9)	C13B—C14B	1.391 (9)
C13A—H13A	0.9500	C13B—H13B	0.9500
C14A—C15A	1.400 (8)	C14B—C15B	1.391 (8)
C14A—H14A	0.9500	C14B—H14B	0.9500
C15A—H15A	0.9500	C15B—H15B	0.9500
C16A—C21A	1.392 (8)	C16B—C17B	1.386 (8)
C16A—C17A	1.396 (9)	C16B—C21B	1.391 (8)
C17A—C18A	1.393 (8)	C17B—C18B	1.388 (8)
C17A—H17A	0.9500	C17B—H17B	0.9500
C18A—C19A	1.386 (9)	C18B—C19B	1.380 (9)
C18A—H18A	0.9500	C18B—H18B	0.9500
C19A—C20A	1.378 (10)	C19B—C20B	1.383 (9)
C19A—H19A	0.9500	C19B—H19B	0.9500
C20A—C21A	1.388 (9)	C20B—C21B	1.400 (8)
C20A—H20A	0.9500	C20B—H20B	0.9500
C21A—H21A	0.9500	C21B—H21B	0.9500
			
C3A—O2A—C2A	114.8 (4)	C3B—O2B—C2B	114.1 (4)
C3A—N1A—C4A	117.2 (5)	C3B—N1B—C4B	118.1 (5)
C3A—N1A—C7A	124.4 (5)	C3B—N1B—C7B	124.0 (4)
C4A—N1A—C7A	118.0 (4)	C4B—N1B—C7B	117.9 (5)
O1A—C1A—C10A	122.5 (5)	O1B—C1B—C10B	121.5 (6)
O1A—C1A—C2A	118.3 (5)	O1B—C1B—C2B	118.9 (5)
C10A—C1A—C2A	119.2 (5)	C10B—C1B—C2B	119.5 (5)
O2A—C2A—C16A	105.8 (4)	O2B—C2B—C16B	106.8 (4)
O2A—C2A—C1A	108.7 (5)	O2B—C2B—C1B	109.2 (5)
C16A—C2A—C1A	109.4 (4)	C16B—C2B—C1B	108.6 (4)
O2A—C2A—H2A	110.9	O2B—C2B—H2B	110.7
C16A—C2A—H2A	110.9	C16B—C2B—H2B	110.7
C1A—C2A—H2A	110.9	C1B—C2B—H2B	110.7
O3A—C3A—N1A	126.2 (5)	O3B—C3B—N1B	126.4 (5)
O3A—C3A—O2A	122.8 (5)	O3B—C3B—O2B	121.8 (5)
N1A—C3A—O2A	110.9 (5)	N1B—C3B—O2B	111.8 (5)
N1A—C4A—C6A	110.4 (5)	N1B—C4B—C5B	112.3 (5)
N1A—C4A—C5A	112.3 (5)	N1B—C4B—C6B	110.9 (5)
C6A—C4A—C5A	112.1 (5)	C5B—C4B—C6B	111.4 (6)
N1A—C4A—H4A	107.2	N1B—C4B—H4B	107.3
C6A—C4A—H4A	107.2	C5B—C4B—H4B	107.3
C5A—C4A—H4A	107.2	C6B—C4B—H4B	107.3
C4A—C5A—H5AA	109.5	C4B—C5B—H5BA	109.5
C4A—C5A—H5AB	109.5	C4B—C5B—H5BB	109.5
H5AA—C5A—H5AB	109.5	H5BA—C5B—H5BB	109.5
C4A—C5A—H5AC	109.5	C4B—C5B—H5BC	109.5
H5AA—C5A—H5AC	109.5	H5BA—C5B—H5BC	109.5
H5AB—C5A—H5AC	109.5	H5BB—C5B—H5BC	109.5
C4A—C6A—H6AA	109.5	C4B—C6B—H6BA	109.5
C4A—C6A—H6AB	109.5	C4B—C6B—H6BB	109.5
H6AA—C6A—H6AB	109.5	H6BA—C6B—H6BB	109.5
C4A—C6A—H6AC	109.5	C4B—C6B—H6BC	109.5
H6AA—C6A—H6AC	109.5	H6BA—C6B—H6BC	109.5
H6AB—C6A—H6AC	109.5	H6BB—C6B—H6BC	109.5
N1A—C7A—C8A	111.7 (5)	N1B—C7B—C9B	113.7 (5)
N1A—C7A—C9A	113.5 (5)	N1B—C7B—C8B	111.6 (5)
C8A—C7A—C9A	112.2 (5)	C9B—C7B—C8B	113.7 (5)
N1A—C7A—H7A	106.3	N1B—C7B—H7B	105.6
C8A—C7A—H7A	106.3	C9B—C7B—H7B	105.6
C9A—C7A—H7A	106.3	C8B—C7B—H7B	105.6
C7A—C8A—H8AA	109.5	C7B—C8B—H8BA	109.5
C7A—C8A—H8AB	109.5	C7B—C8B—H8BB	109.5
H8AA—C8A—H8AB	109.5	H8BA—C8B—H8BB	109.5
C7A—C8A—H8AC	109.5	C7B—C8B—H8BC	109.5
H8AA—C8A—H8AC	109.5	H8BA—C8B—H8BC	109.5
H8AB—C8A—H8AC	109.5	H8BB—C8B—H8BC	109.5
C7A—C9A—H9AA	109.5	C7B—C9B—H9BA	109.5
C7A—C9A—H9AB	109.5	C7B—C9B—H9BB	109.5
H9AA—C9A—H9AB	109.5	H9BA—C9B—H9BB	109.5
C7A—C9A—H9AC	109.5	C7B—C9B—H9BC	109.5
H9AA—C9A—H9AC	109.5	H9BA—C9B—H9BC	109.5
H9AB—C9A—H9AC	109.5	H9BB—C9B—H9BC	109.5
C15A—C10A—C11A	119.1 (5)	C11B—C10B—C15B	119.6 (5)
C15A—C10A—C1A	123.5 (5)	C11B—C10B—C1B	123.4 (5)
C11A—C10A—C1A	117.5 (5)	C15B—C10B—C1B	116.7 (5)
C12A—C11A—C10A	120.8 (6)	C10B—C11B—C12B	119.3 (6)
C12A—C11A—H11A	119.6	C10B—C11B—H11B	120.4
C10A—C11A—H11A	119.6	C12B—C11B—H11B	120.4
C11A—C12A—C13A	119.9 (6)	C13B—C12B—C11B	120.8 (6)
C11A—C12A—H12A	120.0	C13B—C12B—H12B	119.6
C13A—C12A—H12A	120.0	C11B—C12B—H12B	119.6
C12A—C13A—C14A	120.5 (6)	C12B—C13B—C14B	120.4 (6)
C12A—C13A—H13A	119.7	C12B—C13B—H13B	119.8
C14A—C13A—H13A	119.7	C14B—C13B—H13B	119.8
C13A—C14A—C15A	119.4 (6)	C13B—C14B—C15B	119.5 (6)
C13A—C14A—H14A	120.3	C13B—C14B—H14B	120.3
C15A—C14A—H14A	120.3	C15B—C14B—H14B	120.3
C10A—C15A—C14A	120.2 (6)	C14B—C15B—C10B	120.4 (6)
C10A—C15A—H15A	119.9	C14B—C15B—H15B	119.8
C14A—C15A—H15A	119.9	C10B—C15B—H15B	119.8
C21A—C16A—C17A	118.6 (5)	C17B—C16B—C21B	119.7 (5)
C21A—C16A—C2A	122.1 (5)	C17B—C16B—C2B	120.7 (5)
C17A—C16A—C2A	119.3 (5)	C21B—C16B—C2B	119.7 (5)
C18A—C17A—C16A	120.5 (6)	C16B—C17B—C18B	120.1 (6)
C18A—C17A—H17A	119.7	C16B—C17B—H17B	119.9
C16A—C17A—H17A	119.7	C18B—C17B—H17B	119.9
C19A—C18A—C17A	120.2 (6)	C19B—C18B—C17B	120.5 (6)
C19A—C18A—H18A	119.9	C19B—C18B—H18B	119.8
C17A—C18A—H18A	119.9	C17B—C18B—H18B	119.8
C20A—C19A—C18A	119.3 (6)	C18B—C19B—C20B	119.8 (6)
C20A—C19A—H19A	120.4	C18B—C19B—H19B	120.1
C18A—C19A—H19A	120.4	C20B—C19B—H19B	120.1
C19A—C20A—C21A	121.0 (6)	C19B—C20B—C21B	120.1 (6)
C19A—C20A—H20A	119.5	C19B—C20B—H20B	120.0
C21A—C20A—H20A	119.5	C21B—C20B—H20B	120.0
C20A—C21A—C16A	120.3 (6)	C16B—C21B—C20B	119.8 (6)
C20A—C21A—H21A	119.8	C16B—C21B—H21B	120.1
C16A—C21A—H21A	119.8	C20B—C21B—H21B	120.1

### Hydrogen-bond geometry (Å, º)

**Table:**

*D*—H···*A*	*D*—H	H···*A*	*D*···*A*	*D*—H···*A*
C12*A*—H12*A*···O3*A*^i^	0.95	2.36	3.309 (2)	174
C14*B*—H14*B*···O3*B*^ii^	0.95	2.58	3.288 (2)	132
C11*B*—H11*B*···O1*B*^iii^	0.95	2.69	3.553 (2)	152
C21*B*—H21*B*···O1*B*^iii^	0.95	2.62	3.522 (2)	158

Symmetry codes: (i) −*x*+2, *y*+1/2, −*z*; (ii) −*x*+1, *y*+1/2, −*z*+1; (iii) *x*, *y*−1, *z*.
